# Systemic and intrathecal immune activation in association with cerebral and cognitive outcomes in paediatric HIV

**DOI:** 10.1038/s41598-019-44198-z

**Published:** 2019-05-29

**Authors:** C. Blokhuis, C. F. W. Peeters, S. Cohen, H. J. Scherpbier, T. W. Kuijpers, P. Reiss, N. A. Kootstra, C. E. Teunissen, D. Pajkrt

**Affiliations:** 1 Department of Paediatric Haematology, Immunology and Infectious Diseases, Amsterdam University Medical Centers, location Academic Medical Center (AMC), Emma Children’s Hospital, Amsterdam, The Netherlands; 2 Department of Epidemiology & Biostatistics, Amsterdam Public Health Research Institute, Amsterdam University Medical Centers, location VU University Medical Center (VUmc), Amsterdam, The Netherlands; 3 Department of Global Health and Amsterdam Institute of Global Health and Development, Amsterdam University Medical Centers, location AMC, Amsterdam, The Netherlands; 4 HIV Monitoring Foundation, Amsterdam, The Netherlands; 5 Department of Internal Medicine, div. of Infectious Diseases, Amsterdam Institute of Infection and Immunity, Amsterdam University Medical Centers, location AMC, Amsterdam, The Netherlands; 6 Department of Experimental Immunology, Amsterdam University Medical Centers, location AMC, Amsterdam, The Netherlands; 7 Neurochemistry Laboratory and Biobank, Department of Clinical Chemistry, Amsterdam University Medical Centers, location VUmc, Neurocampus, Amsterdam, The Netherlands

**Keywords:** HIV infections, Paediatric research, Neurology

## Abstract

Despite treatment, immune activation is thought to contribute to cerebral injury in children perinatally infected with human immunodeficiency virus (HIV). We aimed to characterize immune activation in relation to neuroimaging and cognitive outcomes. We therefore measured immunological, coagulation, and neuronal biomarkers in plasma and cerebrospinal fluid (CSF) samples of 34 perinatally HIV-infected children aged 8–18 years, and in plasma samples of 37 controls of comparable age, sex, ethnicity, and socio-economic status. We then compared plasma biomarker levels between groups, and explored associations between plasma/CSF biomarkers and neuroimaging and cognitive outcomes using network analysis. HIV-infected children showed higher plasma levels of C-reactive protein, interferon-gamma, interferon-gamma-inducible protein-10, and monocyte chemoattractant protein-1 than controls. In HIV-infected participants, plasma soluble CD14 was positively associated with microstructural white matter (WM) damage, and plasma D-dimer was negatively associated with WM blood flow. In CSF, IL-6 was negatively associated with WM volume, and neurofilament heavy-chain (NFH) was negatively associated with intelligence quotient and working memory. These markers of ongoing inflammation, immune activation, coagulation, and neuronal damage could be used to further evaluate the pathophysiology and clinical course of cerebral and cognitive deficits in perinatally acquired HIV.

## Introduction

Children perinatally infected with human immunodeficiency virus (HIV) show poorer cognitive performance than uninfected peers, even with sustained virological suppression on combination antiretroviral therapy (cART). Widespread neuroimaging abnormalities, including decreased cerebral volume, decreased white matter (WM) integrity, altered neurometabolites, and regional perfusion changes suggest underlying cerebral injury^1–4^. While the underlying mechanisms of paediatric central nervous system (CNS) pathology in treated HIV infection remain unclear, increasing evidence suggests that HIV-associated immune and coagulation activation contribute to morbidity of multiple organ systems, including the CNS^1,5^.

The presence of ongoing inflammation and immune activation in children on long-term cART is suggested by elevated plasma markers of inflammation and monocyte activation, including C-reactive protein (CRP), monocyte chemoattractant protein (MCP-1), and soluble CD14 (sCD14)^6–8^. Further, endothelial activation and a procoagulant state, indicated by increased plasma D-dimer, fibrinogen, soluble intercellular adhesion molecule-1 (sICAM-1), and vascular cell adhesion molecule-1 (sVCAM-1), may facilitate adhesion and migration of immune cells into the brain^7–9^.

Plasma levels of several inflammatory, monocyte, and endothelial markers have already been associated with poorer cognitive functioning in HIV-infected children on suppressive treatment, including CRP, interleukin (IL)-6, sCD14, soluble P-selectin, and fibrinogen^10–12^. However, adult studies show that cerebrospinal fluid (CSF) immunological markers appear to be more closely related to CNS outcomes than plasma markers^13^. CSF MCP-1 and interferon-gamma-inducible protein-10 (IP-10) have been associated with poorer cognitive functioning^14,15^, and MCP-1 and sCD14 with indicators of neuronal injury, such as reduced brain tissue levels of the neurometabolite N-acetylaspartate (NAA), and increased CSF neurofilament light-chain (NFL) levels^16–18^. To date, CSF studies in HIV-infected children remain extremely scarce.

Uniquely for perinatally HIV-infected children, development of both the immune system and the CNS for a large part occurs during exposure to HIV and cART, therefore the consequences of HIV-related immune activation and cerebral changes may well differ from those in adults^19^. As these children are now surviving into adulthood with cART, it is highly important to better understand the underlying mechanisms of CNS pathology to work towards better monitoring, prevention and treatment strategies. To our knowledge, no study has evaluated the relationship between plasma and CSF soluble biomarkers, neuroimaging abnormalities, and cognitive functioning simultaneously in this group.

We therefore aimed to characterize systemic and intrathecal markers of immune activation, endothelial function, and coagulation in a cohort of cART-treated perinatally HIV-infected children, and explore their relations to HIV-associated cognitive and cerebral deficits. First, we compared systemic biomarkers of immune activation, inflammation, endothelial function, and coagulation in HIV-infected children to those in matched uninfected controls. Then, we assessed whether systemic biomarker levels corresponded with intrathecal levels within the HIV-infected group. Lastly, we explored potential relationships between selected markers of immune activation, endothelial function, and previously detected cognitive deficits and magnetic resonance imaging (MRI) abnormalities.

## Methods

### Study participants

This study used cross-sectional data from the first visit of a prospective study focusing on cognitive, cerebral, and retinal injury in paediatric HIV (the NOVICE study, Netherlands Trial Registration ID NTR4074). The study cohort consists of perinatally HIV-infected children between 8–18 years of age, recruited between 2012–2014 from the Emma Children’s Hospital in Amsterdam, and healthy controls. The groups were matched for age, sex, ethnicity and socioeconomic status, and none of the study participants had a history of depression or other psychiatric illnesses or treatments^20^. The study was conducted in accordance with the Helsinki declaration and the study protocol was approved by the medical research ethics committee of the Academic Medical Centre, Amsterdam. Written informed consent was obtained from all legal guardians, and from children aged 12 years of age or above.

### Data collection and biomarker analysis

For the HIV-infected group, data regarding HIV disease history and antiretroviral treatment were obtained from Stichting HIV Monitoring (Netherlands HIV Monitoring Foundation). Neuroimaging was performed on a 3-Tesla MRI (Siemens) using 3D-T1-MPRAGE for volumetric analyses, diffusion tensor imaging for WM diffusivity, magnetic resonance spectroscopy (MRS) for neurometabolite measurements, and arterial spin labelling for cerebral blood flow. Acquisition and processing of neuroimaging data have been detailed previously^2–4^. Cognitive functioning was assessed by a single neuropsychologist using the Wechsler Intelligence Scales for Children (age ≤ 15 years) and Wechsler Adult Intelligence Scales (age ≥ 16 years), and relevant subtests, further detailed in a previous publication by Cohen *et al.*^20^.

Participants underwent venipuncture and a subset of HIV-infected participants underwent a lumbar puncture to obtain blood and CSF for quantification of soluble biomarkers^3^. Within two hours after collection, samples were centrifuged at 1700 × *g* for ten minutes and stored in polypropylene tubes (Sarstedt, Numbrecht, Germany) at −80 degrees Celsius until analysis. We analysed a panel of inflammatory cytokines, chemokines, and endothelial function biomarkers using Meso Scale Discovery, an electrochemiluminescence (ECL)-based immunoassay. Monocyte activation and coagulation markers were quantified using enzyme-linked immunosorbent assays (ELISA), except D-dimer which was measured using a particle-enhanced immunoturbidimetric assay. Serum neurofilament light (NFL) was quantified using a newly developed ECL-based assay for the Meso Scale Discovery platform. In CSF, NFL was quantified using ELISA, neurofilament heavy chain (NFH) using an in-house developed Luminex assay, and total Tau (tTau) using the Innotest (see Supplemental Table 1).

### Statistical analysis

Statistical analysis was performed using Stata 13 (StataCorp, College Station, TX) and R (R Foundation for Statistical Computing, Vienna, Austria). Differences in demographical characteristics between cases and controls were evaluated using Mann-Whitney-U and Chi-square tests.

Prior to analysis, we excluded data from participants with more than 10% missing biomarker measurements. Any values attributable to concentrations below the lower limit of quantification (LLOQ) were imputed by assigning the value of the LLOQ. Biomarkers were excluded from analysis if >30% of values were below the LLOQ. One missing serum NFL value was attributable to a measurement that exceeded the upper LOQ (ULOQ) and was assigned the value of the ULOQ. Both plasma and CSF measurements were log-transformed (by the natural logarithm).

We used a Global test^21^ to assess if the overall plasma-marker expression profile differed between HIV-infected and control groups, adjusted for age and sex. Expression differences of individual plasma markers were evaluated by Mann-Whitney U tests. We performed multiplicity correction (*P*-value adjustment) based on the false discovery rate (FDR), which was controlled at 0.05. To further investigate whether the effects of HIV on plasma-marker expression profile were influenced by sex, we assessed sex-related differences within the HIV-infected group analogously.

The consonance between corresponding plasma and CSF markers was assessed by way of concordance, as operationalized through Kendall’s coefficient of concordance (Kendall’s *W*)^22^. Confidence in the concordance values was evaluated by 95% Bootstrap confidence intervals.

Network analyses were employed to assess the interrelations between markers, clinical characteristics, neuroimaging variables, and indicators of cognitive functioning. Network extraction was based on graphical modelling, using ridge estimation of inverse correlation (i.e., scaled partial correlation) matrices^23^. This estimation technique allows for the number of variables to exceed the number of observations. Resulting networks can be interpreted as conditional independence graphs, i.e., the nodes represent the variables and the edges connecting the nodes represent substantive partial correlations. Linkage in such a graph means that the association between two linked variables cannot be explained away by conditioning on the other variables. The network analyses were performed separately for the plasma and CSF markers, as plasma and CSF represent two different environments. Variables that were selected for the network analyses included 1) a subset of available biomarkers that have previously been shown to be altered in HIV infection and/or may possibly interact with the CNS^1,16,24,25^ 2) age, HIV viral load (VL) and nadir CD4^+^ T-cell Z-score, and 3) neuroimaging and cognitive variables reflecting the HIV-related cognitive impairment and cerebral injury in our cohort (see Supplemental Methods)^2–4,20^.

The datasets generated during and/or analysed during the current study are available from the corresponding author on reasonable request.

### Disclosures

P. Reiss reports fees paid to institution for board membership at Gilead Sciences, Janssen Pharmaceutica, and ViiV healthcare, and grants paid to institution from Gilead Sciences, ViiV Healthcare, Janssen Pharmaceutica, Bristol Myers Squibb, and Merck&Co, outside the submitted work. C. Teunissen reports personal fees for advisory board membership from Fujirebio and Roche, non-financial support (research consumables) from ADxNeurosciences, and has performed contract research or received grants from Janssen Prevention Centre, Boehringer, EIP farma, Roche and Probiodrug, outside the submitted work. D. Pajkrt reports grants paid to institution from AIDS fonds, ViiV Healthcare, Stichting Mitialto, Dr. C.J. Vaillantfonds, and Maarten Kapelle Stichting for the submitted work, and grants paid to institution from TKI Allowance and Emma Foundation, outside the submitted work. The remaining authors have no potential conflicts of interest to disclose.

## Results

### Study participants

Characteristics of the participants are described in Table 1. Of 36 HIV-infected children and 37 uninfected controls initially included in the NOVICE study, we excluded two HIV-infected participants as more than 10% of their marker data was missing. The patient and control groups were similar in terms of age, sex, and ethnicity, and showed no abnormalities on routine physical and neurological examination. HIV was diagnosed at a median age of 2.4 (interquartile range [IQR] 0.7–4.9) years, and 25% had a history of acquired immunodeficiency syndrome (AIDS). The median nadir CD4^+^ T-cell Z-score was −0.7 (reflecting the standard deviation from the age-appropriate norm). Median duration of cART use (85%) was 10.8 years. The subset of patients for whom CSF was available (n = 25) were slightly younger (CSF: 12.9 years; no CSF: 15.1 years; P-value = 0.040), but did not differ regarding other demographic and HIV-related characteristics.

**Table 1:** Study participants.

Demographics		*n*	HIV-infected children	*n*	Uninfected controls	P-value
			*Median (IQR) or n (%)*		*Median (IQR) or n (%)*	
Age at inclusion (years)		34	13.6 (11.5–15.9)	37	12.1 (11.5–15.7)	0.28
Sex (male)		34	16 (47)	37	18 (48)	0.82
Ethnicity (black)		34	25 (74)	37	26 (70)	0.80
BMI (kg/m^2^)		33	18.5 (17.3–21.1)	37	19.6 (17.1–22)	0.57
Blood pressure (mmHg)	*systolic*	33	110 (100–112)	37	105 (95–112)	0.10
	*diastolic*	33	65 (60–72)	37	65 (60–70)	0.82
**HIV**						
Age at HIV diagnosis		34	2.4 (0.7–4.9)			
CDC stage C		34	9 (26)			
CD4^+^ T-cell Z-score	*at nadir*	32	−0.7 (−1.5–0.3)			
	*at inclusion*	34	−0.1 (−0.3–0.2)			
**cART**						
cART	*use at inclusion*	34	29 (85)			
	*age at initiation (years)*	30	2.6 (1.0–5.9)			
Protease inhibitors	*use at inclusion*	34	9 (26)			
	*lifetime exposure (years)*	20	8.1 (5.6–9.5)			
Abacavir	*use at inclusion*	34	14 (41)			
	*lifetime exposure (years)*	27	6.6 (3.2–10.6)			
*Blood HIV RNA* <*150 copies/ml*		34	28 (83)			
*CSF HIV RNA* <*400 copies/ml*		24	20 (83)			

Demographic and clinical characteristics of HIV-infected children and uninfected controls, presented as median (IQR) or n (%), where appropriate.

Definitions: HIV = human immunodeficiency virus; n = number; BMI = body mass index; CDC stage = Centres for Disease Control and Prevention stage (N/A = no/minimal symptoms, B = moderate symptoms, C = severe disease or acquired immunodeficiency syndrome); CD4^+^ T-cell Z-score = standard deviation from age-appropriate mean CD4^+^ T-cell count; cART = combination antiretroviral therapy; CSF = cerebrospinal fluid.

### Immunological and neuronal biomarkers in plasma and csf

#### HIV-infected children compared to controls

Biomarker levels in plasma and CSF are detailed in Table 2. The global test showed an overall significant difference in biomarker profile between HIV-infected and healthy participants (P-value = 0.004), mainly driven by MCP-1. When comparing individual biomarkers between groups, HIV-infected children showed higher plasma levels of MCP-1 (HIV: 99 pg/ml, controls: 72 pg/ml, adjusted P-value = 0.004), interferon-gamma (IFN-γ; HIV: 9.6 pg/ml, controls: 5.7 pg/ml, adjusted P-value = 0.021), IFN-γ-inducible protein-10 (IP-10; HIV: 340 pg/ml, controls: 250 pg/ml, adjusted P-value = 0.035), and CRP (HIV: 0.71 mg/l, controls: 0.28 mg/l, adjusted P-value = 0.035). Biomarker levels were not significantly different between HIV-infected boys and girls (global test: P-value = 0.28; Mann-Whitney-U tests: adjusted P-values > 0.05; data not shown). In a sensitivity analysis comparing only HIV-infected children with undetectable HIV VL to controls, the group difference in IP-10 levels was attenuated (data not shown).

**Table 2:** Inflammation, endothelial activation, neuronal damage and coagulation markers in HIV-infected and uninfected children.

MARKER	HIV-infected children	Uninfected controls	P-value		CSF (HIV-infected)	CONCORDANCE
**Acute phase reactants**	*median (IQR)*	*median (IQR)*	*unadjusted*	*with FDR*	*median (IQR)*	*Kendall’s W (CI)*
*C-reactive protein*	0.71 (0.30–2.48)^*a*^	0.28 (0.16–0.81)^*a*^	0.004*	0.035*	2.38 (0.91–8.40)	**0.99** (0.95–0.99)
*Serum amyloid A*	1.26 (0.65–2.85)^*b*^	1.01 (0.37–1.72)^*b*^	0.13	0.51	—	—
**Interleukins**						
*Interleukin-5*	0.63 (0.46–0.97)	0.72 (0.54–0.95)	0.70	0.84	—	—
*Interleukin-6*	—	—	—	—	0.66 (0.53–0.84)	—
*Interleukin-7*	3.4 (2.3–4.7)	3.0 (1.6–4.6)	0.36	0.75	0.55 (0.29–1.02)	0.46 (0.23–0.68)
*Interleukin-8*	2.2 (1.5–3.2)	2.3 (1.5–3.3)	0.77	0.85	22.9 (15.6–27.8)	0.27 (0.13–0.46)
*Interleukin-10*	0.25 (0.19–0.41)	0.25 (0.18–0.31)	0.62	0.84	0.19 (0.10–0.38)	0.40 (0.23–0.60)
*Interleukin-12 subunit p40*	181 (117–232)	151 (123–203)	0.63	0.84	3.7 (2.3–20.9)	0.49 (0.25–0.76)
*Interleukin-15*	1.6 (1.0–2.0)	1.1 (0.9–1.6)	0.015*	0.10	1.1 (0.9–1.7)	0.52 (0.30–0.75)
*Interleukin-16*	175 (146–226)	189 (158–265)	0.20	0.57	—	—
**Cytokines**						
*Tumor necrosis factor-alpha*	2.3 (1.8–3.3)	2 (1.7–2.4)	0.070	0.32	—	—
*Interferon-gamma*	9.6 (6.9–14.4)	5.7 (4.2–8.7)	0.001*	0.021*	2.29 (1.73–4.20)	0.62 (0.42–0.78)
**Chemokines**						
*Interferon-gamma-inducible protein 10*	340 (239–603)	250 (169–314)	0.004*	0.035*	222 (93–275)	0.49 (0.25–0.74)
*Monocyte chemoattractant protein-1*	99 (77–123)	72 (57–90)	<0.001*	0.004*	225.1 (0.2–347.1)	0.46 (0.25–0.68)
*Monocyte chemoattractant protein-4*	50 (35–60)	46 (28–54)	0.24	0.57	—	—
*Macrophage-derived chemokine*	1273 (1019–1599)	1221 (1034–1722)	0.71	0.84	12.8 (8.4–19)	0.54 (0.31–0.74)
*Thymus and activation regulated chemokine*	90 (49–203)	91 (55–163)	0.89	0.93	2.7 (1.8–5.2)	0.59 (0.36–0.77)
*Macrophage inflammatory protein 1-alpha*	—	—	—	—	8.9 (5.3–13.1)	—
*Macrophage inflammatory protein 1-beta*	49 (38–77)	49 (41–71)	0.64	0.84	12 (9.9–14.3)	**0.71** (0.48–0.87)
*Eosinophil chemotactic protein*	63 (45–87)	56 (37–74)	0.16	0.51	11.3 (5.9–20.3)	0.36 (0.22–0.52)
**Monocyte activation**						
*Soluble cluster of differentiation 14* ^*c*^	1925 (1551–2583)	1861 (1131–2484)	0.26	0.58	42 (30–72)	0.56 (0.34–0.76)
*Soluble cluster of differentiation 163* ^*c*^	216 (150–293)	206 (171–276)	>0.99	>0.99	12.5 (8.6–19.1)	0.50 (0.27–0.74)
**Endothelial activation**						
*Soluble vascular cell adhesion molecule-1* ^*c*^	472 (401–538)	438 (390–503)	0.027*	0.14	0.28 (0.20–0.37)	**0.78** (0.58–0.90)
*Soluble intercellular adhesion molecule-1* ^*c*^	639 (525–838)	591 (437–668)	0.22	0.57	0.71 (0.55–1.23)	**0.78** (0.58–0.92)
**Vascular growth factors**						
*Vascular endothelial growth factor A*	40 (27–67)	50 (35–94)	0.15	0.51	—	—
*Vascular endothelial growth factor C*	81 (61–183)	100 (70–157)	0.42	0.77	—	—
*Vascular endothelial growth factor D*	430 (360–511)	415 (372–504)	0.74	0.84		
*Basic fibroblast growth factor*	2.7 (1.4–10.7)	3 (1.2–5.1)	0.47	0.81	0.36 (0.11–0.99)	0.32 (0.18–0.61)
*FMS-like tyrosine kinase-1 (VEGF receptor-1)*	72 (59–85)	74 (63–90)	0.66	0.84	31 (25–41)	0.54 (0.32–0.74)
*Tyrosine kinase with immunoglobulin-like and EGF-like domains 2 (Angiopoietin-1 receptor)*	4930 (4636–5689)	5267 (4673–5824)	0.50	0.81	16 (14–24)	0.65 (0.42–0.83)
**Coagulation**						
*D-dimer*	0.3 (0.2–0.5)	0.2 (0.2–0.3)	0.23	0.57	—	—
*Von Willebrand factor antigen*	109 (86–148)	107 (87–138)	0.61	0.84	—	—
*Von Willebrand factor propeptide*	97 (84–115)	101 (89–115)	0.69	0.84	—	—
*Thrombin-antithrombin complex*	3.5 (3.1–4.3)	3.5 (3.1–4.2)	0.90	0.93	—	—
*Prothrombin fragment 1 + 2*	126 (108–153)	115 (101–151)	0.39	0.75	—	—
**Neuronal injury**						
*Neurofilament heavy chain*	—	—	—	—	124 (99–149)	—
*Total Tau protein*	—	—	—	—	158 (102–235)	—

Plasma and CSF concentrations are displayed as median (interquartile range) in pg/ml, unless specified otherwise. Coagulation biomarkers were only measured in plasma, and neuronal markers NFH and Tau only in CSF. Markers were excluded from analysis when less than 70% of measurements fell within the range of quantification (as specified in the Supplemental Methods). Plasma markers were compared between HIV-infected children (n = 34) and uninfected controls (n = 37) using the Mann-Whitney-U test, and P-values are displayed before and after multiplicity correction by FDR control. Concordance between plasma and CSF levels (sCD14/sCD163 n = 25, other biomarkers n = 23) in the HIV-infected group was assessed using Kendall’s coefficient of concordance (Kendall’s W).

*Definitions*: HIV = human immunodeficiency virus; CSF = cerebrospinal fluid; FDR = False Discovery Rate; Kendall’s W = Kendall’s coefficient of concordance; CI = bootstrap confidence interval. ^a^mg/l; ^b^µg/l; ^c^ng/ml; *P-value < 0.05.

#### Plasma-CSF concordance in HIV-infected children

The concordances between corresponding plasma and CSF biomarkers show considerable variation (Table 2). We observed the highest concordance coefficients for CRP (W = 0.99), sICAM-1 (W = 0.78), sVCAM-1 (W = 0.78), and macrophage inflammatory protein-1-beta (MIP-1β; W = 0.71).

### Associations between biomarkers, cerebral injury and cognitive performance in hiv-infected children

**Figure 1:**
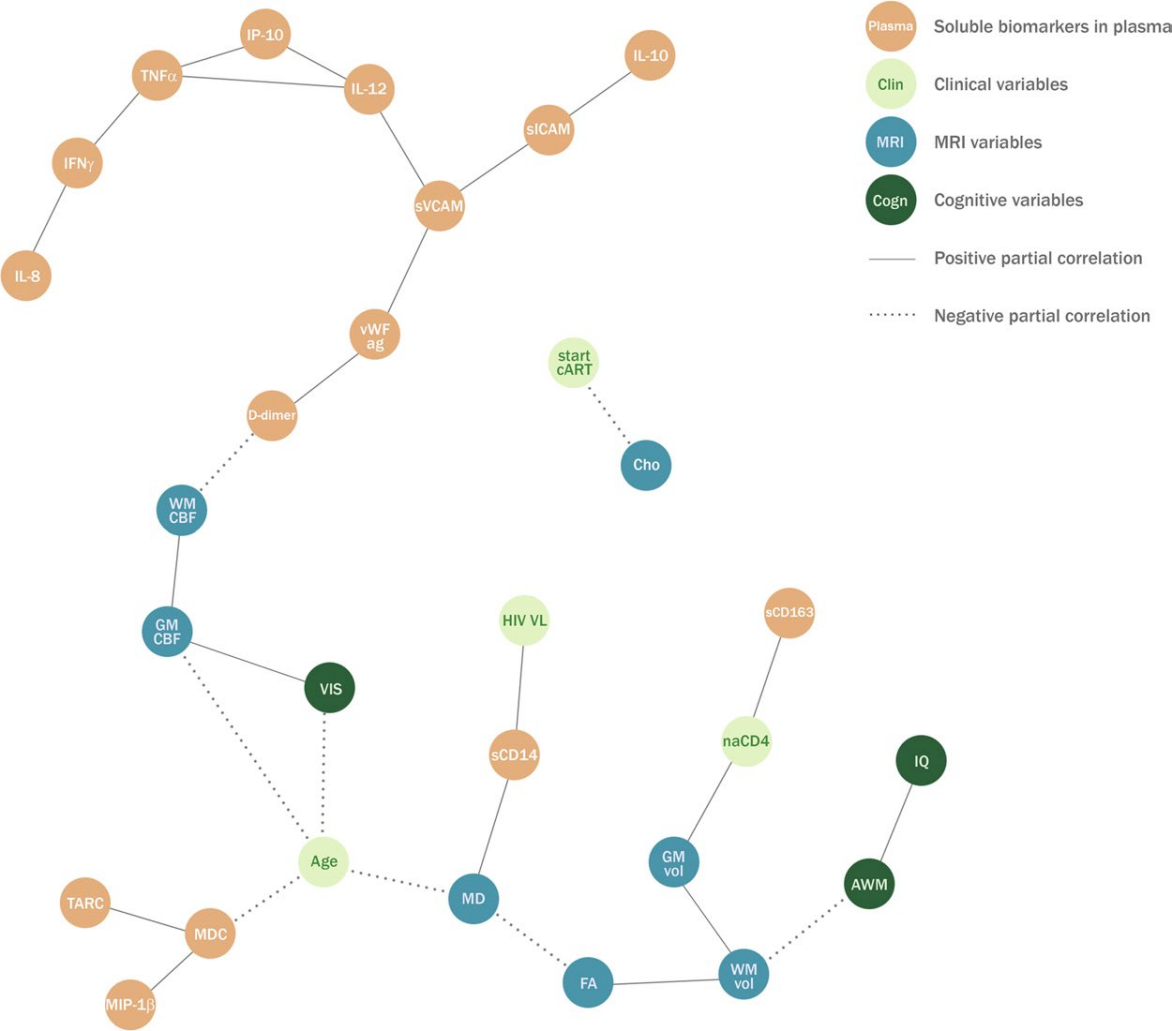
Associations between plasma soluble biomarkers, clinical characteristics, neuroimaging, and cognitive functioning. Retained network visualizing partial correlations between plasma biomarkers, clinical characteristics, neuroimaging variables and cognitive functioning in 21 HIV-infected participants. Each node represents a variable. The solid edges represent positive partial correlations and the dashed edges represent negative partial correlations. For example, the connection between visuomotor performance (VIS) and gray matter cerebral blood flow (GM CBF) can be interpreted as a positive association between these two variables that cannot be explained by any of the other variables in the model, such as intelligence quotient (IQ) or age. Further, the connection between HIV viral load (HIV VL) and sCD14 also means that if you take sCD14 into account, there is no significant correlation between HIV VL and mean white matter diffusivity (MD). The following variables were included in the analysis, but were not retained in the final network: C-reactive protein, interleukin-15, monocyte chemoattractant protein-1, subcortical cerebral blood flow, prothrombin fragment 1 + 2, and processing speed. Definitions: AWM = attention/working memory; Cho = white matter choline-to-creatine ratio; FA = fractional anisotropy; GM CBF = grey matter cerebral blood flow; GM vol = grey matter volume; HIV = human immunodeficiency virus; IFNγ = interferon-gamma; IL = interleukin; IL-12 = interleukin 12p40; IP-10 = interferon-gamma-inducible-protein-10; IQ = intelligence quotient; MD = mean diffusivity; MDC = macrophage-derived chemokine; MIP = macrophage inflammatory protein; MRI = magnetic resonance imaging; naCD4 = nadir CD4^+^ T-cell count Z-score; TNFα = tumor necrosis factor-alpha; sCD = soluble cluster of differentiation; sICAM-1 = soluble intercellular adhesion molecule-1; start cART = age at which combination antiretroviral therapy was initiated; sVCAM-1 = soluble vascular cell adhesion molecule-1; TARC = thymus and activation regulated chemokine; VIS = visuomotor integration; VL = viral load; vWF-ag = von Willebrand factor antigen; WM CBF = white matter cerebral blood flow; WM vol = white matter volume.

**Figure 2:**
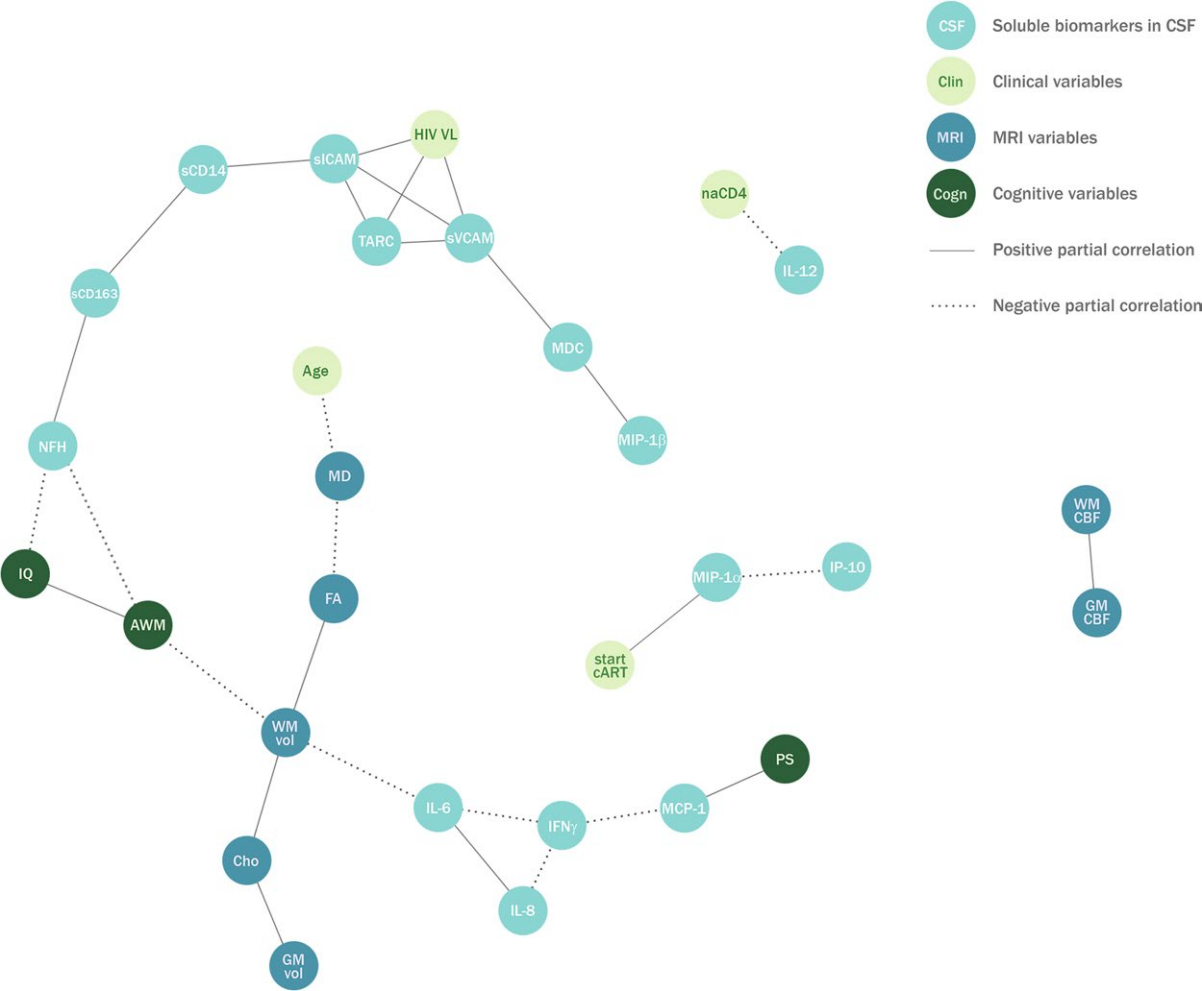
Associations between CSF soluble biomarkers, clinical characteristics, neuroimaging, and cognitive functioning. Retained network visualizing partial correlations between cerebrospinal fluid biomarkers, clinical characteristics, neuroimaging variables and cognitive functioning in 13 HIV-infected participants. Each node represents a variable. The solid edges represent positive partial correlations and the dashed edges represent negative partial correlations. For example, the connection between interleukin-6 (IL-6) and white matter volume (WM vol) can be interpreted as a negative association between these two variables that cannot be explained by any of the other variables in the model, such as gray matter volume (GM vol) or age. Further, the connection between monocyte-chemoattractant protein-1 (MCP-1) and processing speed (PS) also means that if you take MCP-1 into account, there is no significant correlation between processing speed and interferon-gamma (IFNγ). The following variables were included in the analysis, but were not retained in the final network: C-reactive protein, IL-10, IL-15, total Tau protein, subcortical cerebral blood flow, and visuomotor integration. Definitions: AWM = attention/working memory; CSF = cerebrospinal fluid; Cho = white matter choline-to-creatine ratio; FA = fractional anisotropy; GM CBF = grey matter cerebral blood flow; GM vol = grey matter volume; HIV = human immunodeficiency virus; IFNγ = interferon-gamma; IL = interleukin; IL-12 = interleukin 12p40; IP-10 = interferon-gamma-inducible-protein-10; IQ = intelligence quotient; MCP-1 = monocyte chemoattractant protein; MD = mean diffusivity; MDC = macrophage-derived chemokine; MIP = macrophage inflammatory protein; MRI = magnetic resonance imaging; naCD4 = nadir CD4^+^ T-cell count Z-score; NFH = neurofilament heavy-chain; sCD = soluble cluster of differentiation; sICAM-1 = soluble intercellular adhesion molecule-1; start cART = age at which combination antiretroviral therapy was initiated; sVCAM-1 = soluble vascular cell adhesion molecule-1; TARC = thymus and activation regulated chemokine; VL = viral load; vWF-ag = von Willebrand factor antigen; WM CBF = white matter cerebral blood flow; WM vol = white matter volume.

For the network analyses, we included 21 cases with complete data (on the variables of interest) for the plasma-biomarker network (Fig. 1), and 13 cases for the CSF-biomarker network (Fig. 2). Here, we focus on describing conditional associations of biomarkers with HIV-related, neuroimaging, and cognitive outcomes.

#### Associations between soluble biomarkers and HIV characteristics

Plasma sCD14 levels were positively associated with HIV VL (Fig. 1). Plasma sCD163 levels were positively associated with the nadir CD4+T-cell Z-score. In CSF (Fig. 2), thymus and activation regulated chemokine (TARC), sICAM-1, and sVCAM-1 were positively associated with higher CSF HIV VL. CSF IL-12p40 was negatively associated with the nadir CD4+T-cell Z-score, and CSF MIP-1-alpha was positively associated with age at cART initiation.

#### Associations between soluble biomarkers and cerebral/cognitive outcomes

Plasma sCD14 was positively associated with higher WM mean diffusivity (MD; Fig. 1). Plasma D-dimer levels were negatively associated with WM cerebral blood flow (CBF). CSF IL-6 levels were negatively associated with WM volume (Fig. 2). CSF NFH was negatively associated with intelligence quotient (IQ) and attention/working memory. CSF MCP-1 was positively associated with processing speed. Of note, no conditional associations were retained between CRP, IP-10, IFN- γ and the included cerebral or cognitive outcomes.

## Discussion

In this study, HIV-infected children showed higher systemic levels of CRP, IFN-γ, IP-10, and MCP-1 compared to matched healthy controls, indicating ongoing low-grade immune activation and inflammation despite having suppressed HIV VL levels in plasma and CSF. Concordance between systemic and intrathecal biomarker levels was limited, suggesting that patterns of immune activation and inflammation may differ between these two compartments. Several immunological and neuronal markers, including plasma sCD14, CSF IL-6, and CSF NFH, were associated with poorer cerebral and cognitive outcomes, which may imply that immune activation and neurodegeneration play a role in paediatric HIV-associated cerebral dysfunction.

The pattern of systemic immune activation (with increased CRP, IFN-γ, IP-10, and MCP-1) shows consistency with previous reports. Elevations of systemic MCP-1, a potent driver of monocyte migration into the CNS, and CRP were previously reported in cART-treated HIV-infected children and adolescents^6,7^. Elevated plasma and CSF IP-10 levels have been reported in adults suppressed on cART^25^.

MCP-1 and IP-10 have been previously implicated in HIV-associated neurocognitive disease in cART-treated adults, with associations between increased CSF MCP-1 and IP-10 levels and the presence or severity of cognitive impairment^14,15^, as well as MRS alterations indicative of neuronal damage and glial proliferation^17,18^. In our study, we found no associations between elevated plasma CRP, MCP-1, IFN-γ, or IP-10, and cerebral or cognitive outcomes. In the CSF network, MCP-1 was positively associated with processing speed, while no associations were retained between CRP, IFN-γ, or IP-10, and any of the cognitive or cerebral outcomes. Several other adult and paediatric studies were also unable to confirm an association between MCP-1 in plasma or CSF and cognitive or neurological impairment^11,25,26^.

These discrepant findings may have both pathophysiological and methodological explanations. First, we were unable to determine if MCP-1 levels in CSF are elevated in the HIV-infected group as elevated plasma MCP-1 showed poor concordance with CSF levels, and we had no CSF available from the healthy controls. However, even elevated CSF MCP-1 was not consistently associated with cognitive outcomes in other studies^25^, suggesting a relationship between increased levels of MCP-1 and CNS injury may require specific conditions. One theory states that MCP-1 alters the permeability of the blood-brain barrier and thereby increase macrophage migration into the brain, but does not attract CD14+ CD16+ monocytes, which are associated with high risk of HIV-associated neurocognitive disorders but do not express the relevant chemokine C-C motif receptor 2 (CCR2) that binds MCP-1^27^. Their migration into the brain may thus co-depend on activation by other factors such as soluble P-selectin, which was not measured in the current study but has been previously associated with poorer cognitive functioning in HIV-infected youth^11,28^. Additionally, previously detected relationships between MCP-1 and CNS outcomes in adults appear to be enhanced in the presence of viral replication^29,30^. Since the large majority of our study participants had undetectable HIV VL in blood and CSF, the number of participants with a detectable HIV plasma and CSF VL in our study may be too small to robustly detect associations with MCP-1 or influence its relationship with other outcomes.

Some of the variation may also suggest a difference in the pathogenesis of HIV-associated cognitive impairment between adults and children, which strongly differ in terms of participant characteristics (e.g., age at diagnosis, lifestyle factors, co-morbidities). Inherently, study protocols also differ (e.g., tests used to measure cognitive functioning, criteria used to define cognitive impairment) which should be taken into account when interpreting the results. This stresses the importance of further research to evaluate the role of MCP-1 and other immunological biomarkers in HIV-associated CNS disease specifically in the paediatric population, as well as potential differences in pathogenesis between children and adults. Finally, most other studies evaluating biomarkers in relation to CNS outcomes adjust for demographical and HIV-related variables, but generally not as extensively for other soluble biomarkers as the network analysis in the current study. This provides a unique insight into the interplay between many factors of potential influence on HIV-associated CNS pathology, and should be taken into account when comparing findings with other studies.

Plasma levels of sCD14 and sCD163 have previously been reported to be increased in HIV-infected children^8,31^, but these markers were not significantly elevated in the HIV-infected group in our study. This could (in part) be due to long term HIV VL suppression, which has been shown to reduce plasma sCD14 and sCD163 levels over time^31,32^. In our study, plasma sCD163 was positively associated with nadir CD4+ T-cell Z-scores. This association may reflect that children with less severe immune suppression may have been exposed to HIV replication for a longer period prior to starting cART, although we did not find direct evidence for this in the current study and this should be investigated further.

Plasma sCD14, a measure of gut microbial translocation and monocyte activation^33^, was associated with plasma HIV VL and with increased WM diffusivity, a measure of poorer myelin integrity. The myelination process (indicated by increase in fractional anisotropy [FA] and decrease in mean diffusivity [MD] on diffusion tensor imaging) continues well into the second decade of life^34^, which is consistent with the negative association between age and MD in our study (Figs 1 and 2). Previous studies in HIV-infected children reported a relationship between increased MD and plasma HIV VL^35,36^. However, in our study, HIV VL and MD are independent given sCD14, which may imply that microbial translocation and/or monocyte activation better predict microstructural WM injury than HIV VL. Higher levels of IL-6 in CSF were associated with reduced WM volume. As WM volume was consistently positively associated with FA in this study (as shown in Figs 1 and 2), it might indicate a potential relationship between inflammation and poorer myelination^37^. In CSF, HIV VL was related to higher levels of endothelial activation markers sVCAM-1 and sICAM-1, but CSF HIV VL and sCD14 were independent given sICAM-1 (as shown in Fig. 2). This might indicate that unsuppressed HIV in the CNS promotes endothelial activation, and that the latter further increases adhesion and migration of activated monocytes across the blood-brain barrier^38^.

The CSF network also showed an association between higher sCD163 and NFH, which is consistent with findings in adults with HIV-associated cognitive impairment^16^, and could indicate a relationship between monocyte activation and axonal damage. We also found an association between CSF NFH levels and poorer IQ and attention/working memory. Correlations between CSF NFH and cognitive impairment were also found in cART-treated adults, but in different cognitive domains (processing speed and memory)^16^. CSF NFL was not correlated with cognitive impairment in that study, possibly because NFH is more stable and protease resistant^39^. In our study, NFL was excluded from analyses as both blood and CSF measurements fell below the limit of detection in >30% of the children, suggesting it only reflects neuroaxonal injury in a subset of cART-treated HIV-infected children. Considering the superior stability of CSF NFH and the detected associations with cerebral and cognitive outcomes, we hypothesize it may be a more sensitive marker to evaluate neuroaxonal injury in this population.

CSF levels of the chemokine MIP-1α were positively associated with the age at which cART was initiated, but not with any specific CNS outcomes. MIP-1α has been previously reported to be elevated in CSF of aviremic HIV-infected adults, regardless of cognitive status^25^. Considering the association with age at cART initiation in our study, CSF MIP-1α could be a potentially interesting inflammatory marker to study in relation to viral reservoirs in the CNS.

In previous studies, nadir CD4^+^ T-cell counts have been associated with cerebral and cognitive deficits in perinatally HIV-infected children^3,35,40^. In our multivariable network analyses, only a single association between the nadir CD4^+^ T-cell Z-score and grey matter (GM) volume was retained in the plasma network (Fig. 1). This association is in line with earlier findings^2^, but was not present in the CSF network (Fig. 2). Overall, (markers of) immune activation and inflammation seem to be associated more frequently and strongly with CNS injury in cART-treated children than conventional HIV VL measurements or (nadir) CD4^+^ T-cell counts in our study.

The negative association between plasma D-dimer and WM CBF may suggest that a procoagulant state may affect the cerebral vasculature in paediatric HIV. While we have no direct measures of cerebrovascular structure and function available in our cohort, several systemic factors known to be associated with cerebrovascular disease^41^ have been reported in perinatally HIV-infected children. These include increased plasma CRP^6^, reduced flow mediated dilatation of the brachial artery^42^, and increased carotid intima media thickness^43^. Since the vascular disease process leading to atherosclerosis begins in childhood, and progression with age may be additionally modified by HIV^44^, these measurements and other traditional cerebrovascular risk factors are important to follow-up longitudinally in paediatric HIV.

While our study uniquely evaluates plasma and CSF biomarkers in combination with MRI and cognitive outcomes in perinatally HIV-infected children, it is subject to several limitations. Our sample size was relatively small, as CSF was available in a subset of children only, and MRI data was not always complete due to contraindications or scanning artifacts^2–4^. This may have led to insufficient power for the detection of (subtle) differences and associations. Data on several prenatal and early life factors known to influence brain development, such as maternal health during pregnancy, preterm birth, and malnutrition^19,45,46^, were not available in the current study. The cross-sectional setup of the study prevents drawing causal conclusions, and we are thus unable to definitively distinguish between inflammation as cause or consequence of cerebral injury. Our study findings should therefore be regarded as exploratory and hypothesis-generating.

In conclusion, our study shows ongoing immune activation and inflammation in perinatally HIV-infected children, despite virological suppression with cART. While biomarker changes were subtle, and patterns differed between plasma and CSF, several markers of inflammation (IL-6), microbial translocation and monocyte activation (sCD14), coagulation (D-dimer), and neuronal damage (NFH) were associated with markers of WM brain perfusion, WM injury and poorer cognitive outcomes. These results implicate a role for neuroinflammation and –degradation in paediatric HIV-related CNS disease. In accordance with previous studies, no single biomarker appears suitable to replace neuroimaging or neuropsychological testing in children treated for perinatally acquired HIV. Therefore, to increase our understanding of the underlying neuropathogenesis of HIV-associated cerebral and cognitive injury, future studies should expand to larger study groups and longitudinal research settings, focusing on the relationships between these markers and vascular structure and function, neuroimaging, and cognitive outcomes over time. Continued research could provide a framework for the identification of biochemical and neuroimaging markers, or a combination thereof, to monitor or predict cognitive decline in this population. A better understanding of the relationship between neuroinflammation and cognitive function in paediatric HIV may also provide opportunities to study adjuvant treatment options, that may protect or even improve cognitive function in perinatally HIV-infected children.

## Supplementary information

Supplemental methods
