# Supplementary material for: Systemic and intrathecal immune activation in association with cerebral and cognitive outcomes in paediatric HIV
Source: Sci Rep. 2019 May 29;9:8004. doi: 10.1038/s41598-019-44198-z (PMC6541601; doi:10.1038/s41598-019-44198-z)
Supplement: Supplementary file 1 — Supplemental methods [file 41598_2019_44198_MOESM1_ESM.pdf]

Systemic and intrathecal immune activation in association with cerebral and cognitive outcomes in paediatric HIV

C. Blokhuis[1], C.F.W. Peeters[2], S. Cohen[1], H.J. Scherpbier[1], T.W. Kuijpers[1], P. Reiss[3-5], N.A. Kootstra[6], C.E. Teunissen[7], and D. Pajkrt[1]

1 Department of Paediatric Haematology, Immunology and Infectious Diseases, Amsterdam University Medical Centers, location Academic Medical Center (AMC), Emma Children's Hospital, Amsterdam, the Netherlands

2 Department of Epidemiology & Biostatistics, Amsterdam Public Health Research Institute, Amsterdam University Medical Centers, location VU University Medical Center (VUmc), Amsterdam, The Netherlands

3 Department of Global Health and Amsterdam Institute of Global Health and Development, Amsterdam University Medical Centers, location AMC, Amsterdam, the Netherlands

4 HIV Monitoring Foundation, Amsterdam, the Netherlands

5 Department of Internal Medicine, div. of Infectious Diseases, Amsterdam Institute of Infection and Immunity, Amsterdam University Medical Centers, location AMC, Amsterdam, the Netherlands

6 Department of Experimental Immunology, Amsterdam University Medical Centers, location AMC, Amsterdam, the Netherlands

7 Neurochemistry Laboratory and Biobank, Department of Clinical Chemistry, Amsterdam University Medical Centers, location VUmc, Neurocampus Amsterdam, the Netherlands

Supplemental materials

- Supplemental Table 1: Biomarker quantification
- Supplemental Methods

Table 1. Biomarker quantification

Biomarkers		Method	Dynamic range		Available for analysis	
			Plasma	*CSF*	*Plasma*	*CSF*
Acute phase reactants						
C-reactive protein	CRP	MSD	1.21-216500	11.2-216500	✓	✓
Serum amyloid A	SAA	MSD	18.1-267000	64.7-267000	✓	<70%
Interleukins						
Interleukin-1-alpha	IL-1a	MSD	0,082-332	0.031-321	<70%	<70%
Interleukin-1-beta	IL-1b	MSD	0,03-510	0.439-2200	<70%	<70%
Interleukin-2	IL-2	MSD	0.232-1470	0.114-1470	<70%	<70%
Interleukin-4	IL-4	MSD	0.038-216	0.047-216	<70%	<70%
Interleukin-5	IL-5	MSD	0.107-780	0.034-720	✓	<70%
Interleukin-6	IL-6	MSD	0.108-769	0.138-769	<70%	✓
Interleukin-7	IL-7	MSD	0.165-637	0.057-648	✓	✓
Interleukin-8 (CXCL8)	IL-8	MSD	0.086-507	0,09-507	✓	✓
Interleukin-10	IL-10	MSD	0.061-324	0,06-324	✓	✓
Interleukin-12 subunit p40	IL-12p40	MSD	0.502-2720	0.153-2370	✓	✓
Interleukin-13	IL-13	MSD	1.03-530	1.17-769	<70%	<70%
Interleukin-15	IL-15	MSD	0.192-654	0.049-662	✓	✓
Interleukin-16	IL-16	MSD	1.47-2230	0.439-2200	✓	<70%
Cytokines						
Tumor necrosis factor-alpha	TNF-a	MSD	0.115-316	0.119-316	✓	<70%
Tumor necrosis factor-beta	TNF-b	MSD	0.089-546	0.023-546	<70%	<70%
Interferon-gamma	IFN-g	MSD	1.13-1410	1.23-1410	✓	✓
Granulocyte macrophage colony-stimulating factor	GM-CSF	MSD	n/a	1.9-750	n/a	<70%
Chemokines						
Interferon-gamma-inducible protein 10 (CXCL10)	IP10	MSD	0.138-2380	0.203-2380	✓	✓
Monocyte chemoattractant protein-1 (CCL2)	MCP-1	MSD	0.109-471	0.151-471	✓	✓
Monocyte chemoattractant protein-4 (CCL13)	MCP-4	MSD	3.08-588	3.98-588	✓	<70%
Macrophage-derived chemokine (CCL22)	MDC	MSD	3.96-9500	5.6-9500	✓	✓
Thymus and activation regulated chemokine (CCL17)	TARC	MSD	0.133-1430	0.401-1430	✓	✓
Macrophage inflammatory protein 1-alpha (CCL3)	MIP-1a	MSD	3.44-991	5,27-991	<70%	✓
Macrophage inflammatory protein 1-beta (CCL4)	MIP-1b	MSD	0.545-1020	2.43-1020	✓	✓
Eosinophil chemotactic protein (CCL11)	Eotaxin	MSD	4.11-1380	5.41-1380	✓	✓
Eosinophil chemotactic protein-3 (CCL26)	Eotaxin-3	MSD	2.78-4370	2.85-4370	<70%	<70%
Monocyte activation						
Soluble cluster of differentiation 14	sCD14	ELISA	62.5-4000	62.5-4000	✓	✓
Soluble cluster of differentiation 163	sCD163	ELISA	156-10000	156-10000	✓	✓
Endothelial activation						
Soluble vascular cell adhesion molecule-1	sVCAM-1	MSD	3.88-53500	17.8-53500	✓	✓
Soluble intercellular adhesion molecule-1	sICAM-1	MSD	1.61-56500	6.52-56500	✓	✓
Vascular growth factors						
Vascular endothelial growth factor A	VEGF-A	MSD	1.31-966	0.25-894	✓	<70%
Vascular endothelial growth factor C	VEGF-C	MSD	11.2-23500	12-23500	✓	<70%
Vascular endothelial growth factor D	VEGF-D	MSD	2.04-22850	5.25-22850	✓	<70%
Basic fibroblast growth factor	bFGF	MSD	0.087-1840	0.108-1840	✓	✓
FMS-like tyrosine kinase-1 (VEGF receptor-1)	Flt-1	MSD	1.22-8300	1.17-8300	✓	✓
Tyrosine kinase with immunoglobulin-like and EGF-like domains 2 (Angiopoietin-1 receptor)	Tie2	MSD	10.1-79000	13.7-79000	✓	✓
Coagulation						
D-dimer	D-dimer	Innovance[1]	0.05-4.46[a]	n/a	✓	n/a
Von Willebrand factor antigen	vWF-ag	ELISA[b]	n/a	n/a	✓	n/a
Von Willebrand factor propeptide	vWF-pro	ELISA[b]	n/a	n/a	✓	n/a
Thrombin-antithrombin III complex	TAT	Enzygnost[2]	2-60[a]	n/a	✓	n/a
Prothrombin fragment 1+2	F1+2	Enzygnost[3]	20-1200[c]	n/a	✓	n/a
Neuronal injury						
Neurofilament light chain	NFL	MSD/ELISA[a]	7.8-5000	100-10000	<70%	<70%
Neurofilament heavy chain	NFH	Luminex[4]	n/a	19.5-5000	n/a	✓
Total Tau protein	tTau	Innotest[5]	n/a	52-1200	n/a	✓

All analyzed biomarkers with quantification methods and dynamic ranges according to the manufacturers. Biomarkers were excluded for further analyses if less than 70% of measured values fell within the range of quantification.

Definitions: CSF=cerebrospinal fluid; MSD=Meso Scale Discovery[6]; ELISA=enzyme-linked immunosorbent assay; n/a=not available.

Footnotes: Footnotes: a=mg/l; b=determined on in-house developed ELISA, measured as percentage; c=pmol/l; d=CSF NFL was quantified using ELISA, and serum NFL was quantified with an in-house developed kit for Meso Scale Discovery, utilizing the same antibodies as the ELISA. [7]

References for Table 1:

1. Food and Drug Administration. *INNOVANCE™ D-Dimer: 510(k) Substantial Equivalence Determination Decision Summary*. Available at: https://www.accessdata.fda.gov/cdrh_docs/reviews/K081732.pdf

2. Dade Behring. *Enzyme Immunoassay for the Determination of Human Thrombin/ Antithrombin III Complex*. 1998. Available at: http://www.medcorp.com.br/medcorp/upload/downloads/Complexo Enzim tico Trombina e Antitrombina_2006329978.PDF

3. Food and Drug Administration. *Enzygnost™ F1+2 (Monoclonal): 510(k) Substantial Equivalence Determination Decision Summary*. Available at: https://www.accessdata.fda.gov/cdrh_docs/reviews/K042687.pdf

4. Koel-Simmelink MJA, Vennegoor A, Killestein J, et al. The impact of pre-analytical variables on the stability of neurofilament proteins in CSF, determined by a novel validated SinglePlex Luminex assay and ELISA. *J Immunol Methods*. 2013;402(1-2):43-49.

5. Van Der Flier WM, Pijnenburg YA, Prins N, et al. Optimizing patient care and research: The Amsterdam dementia cohort. *J Alzheimer's Dis*. 2014;41(1):313-327.

6. Meso Scale Discovery. *High Performance Biomarker Assays and Services. Singleplex and Multiplex Assay List.* 2017. Available at: https://www.mesoscale.com/~/media/files/handouts/assaylist.pdf

7. Gaiottino J, Norgren N, Dobson R, et al. Increased Neurofilament Light Chain Blood Levels in Neurodegenerative Neurological Diseases. Reindl M, ed. *PLoS One*. 2013;8(9):1-9.

Supplemental Methods

This study includes data concerning 36 perinatally human immunodeficiency virus (HIV)-infected children and 37 uninfected controls. Interest lies with exploring inflammation and immune activation as measured through plasma and cerebrospinal fluid (CSF) markers. It was previously found that HIV-infected children display neuroimaging abnormalities and impaired cognitive functioning in comparison to controls. Four questions directed the analyses:

(1) Are the plasma markers differentially expressed between HIV-infected children and controls?

(2) Are the plasma markers concordant with their corresponding CSF markers?

(3) Can we characterize, for HIV-infected children, associations between HIV-related measurements and plasma/CSF markers?

(4) Can we characterize, for HIV-infected children, associations between plasma/CSF markers and neuroimaging abnormalities/cognitive functioning measurements?

Data preprocessing

Plasma markers were measured in all study participants. Prior to analysis, we removed two cases due to >10% missing values (due to unavailable plasma samples for those measurements). For further analyses, we had plasma-marker data available from 34 HIV-infected children and 37 controls. CSF of 25 HIV-infected participants was available for soluble cluster of differentiation (sCD)14 and sCD163 measurements, and of 23 participants for the remaining biomarker measurements.

We then identified plasma and CSF measurements that were listed as below the lower limit of quantification (LLOQ). Biomarkers were excluded from analysis if >30% of values were below the LLOQ. Remaining values below the LLOQ were imputed by assigning the value of the LLOQ as indicated by the manufacturer (Supplemental Table 1). One missing value of serum NFL was attributable to a value exceeding the upper LOQ, and was thus assigned the value of the upper LOQ. All markers were then log-transformed using the natural logarithm.

Approach to question 1.

Question 1 asked if there were (subsets of) plasma markers that were differentially expressed between HIV-infected children and controls. A two-tier approach was considered:

Most plasma features were termed approximately normally-distributed on the ln-scale. As the features displayed some non-normality and as the group sizes differed (implying unequal group variances) a nonparametric alternative to the independent samples t-test was used: the Mann-Whitney U test. This test was employed to each feature (i.e., plasma marker) for the juxtaposition of interest. (When possible) Exact p-values were calculated for each test instance. Multiplicity correction (p-value adjustment) was performed based on the false discovery rate (FDR), which was controlled at .05.

In addition, global differential expression testing was performed. Specifically, a Global Test was employed.[1] This test may be seen as a method that looks for differentially expressed feature-sets. It can be used for testing the overall expression profile to calculate whether it is notably different between conditions. This test is suitable when there may be insufficient or low power to detect individual plasma markers. This test may then indicate/detect if the overall plasma-marker expression profile differs markedly between the HIV and control conditions. Moreover, this test may take possible confounders into account. The Global Test provides a single p-value for a chosen feature set. The chosen feature set is the set of all (retained) plasma markers (33 features).

These two approaches were considered for complementarity. The first approach was a standard approach. The second approach is lesser known, but could have advantages in situations of low-power. In addition, consistency in findings over these two approaches (in terms of the differential expression signature) strengthen the findings. The possible confounders that were considered in the Global Testing exercise were age at inclusion and gender.

Approach to question 2.

Question 2 asked if the plasma markers were concordant with their corresponding CSF markers. This question thus concerned markers that, within the same person, were measured in both plasma and CSF. This resembles a test-retest setting. Hence, the notion of concordance was appropriate to assess the consonance between corresponding plasma and CSF markers. Here, concordance was operationalized with Kendall's W.[2] We constructed 95% Bootstrap confidence intervals around the concordance values. The number of Bootstrap iterations was set to 10,000.

Approach to questions 3 and 4

To explore associations between soluble biomarkers, clinical characteristics, neuroimaging, and cognitive functioning, we assessed conditional associations as measured through partial correlations.[3] The non-zero partial correlations between these variables form a network that can be interpreted as a conditional independence graph. Linkage in such a network implies that variables, conditional on the effects of all other variables, are associated. Hence, linkage means that the association between two linked variables cannot be explained by conditioning on the other variables.

The variables selected for the network analyses included the following:

1) Biomarkers representing immune activation, inflammation, endothelial function, and neuronal damage in relation to HIV.[4–7] Available markers were slightly different per compartment (see also Supplemental Table 1):

    a. **Plasma:** C-reactive protein (CRP), IL-8, IL-10, IL-12p40, IL-15, tumor necrosis factor-alpha (TNFα), interferon-gamma (IFNγ), IFNγ-inducible protein-10 (IP-10), monocyte chemoattractant protein-1 (MCP-1), macrophage inflammatory protein (MIP)-1β, macrophage-derived chemokine (MDC), thymus and activation regulated chemokine (TARC), tumor necrosis factor-alpha (TNFα), soluble cluster of differentiation (sCD)14, sCD163, soluble vascular cell adhesion molecule-1 (sICAM-1), soluble intercellular adhesion molecule-1 (sVCAM-1), D-dimer, von Willebrand factor antigen (vWF ag), prothrombin fragment 1 and 2 (F1+2).

    b. **CSF:** CRP, IL-6, IL-8, IL-10, IL-12p40, IL-15, IFNγ, IP-10, MCP-1, MIP-1α, MIP-1β, MDC, TARC, sCD14, sCD163, sICAM-1, sVCAM-1, neurofilament heavy-chain (NFH), total Tau protein (Tau).

2) Clinical variables: age, as important factor concerning brain and cognitive development; HIV viral load (VL) at study inclusion; $CD4^+$ T-cell count Z-score at nadir, which represents the standard deviation from the age-appropriate mean at its lowest point, before or shortly after cART was initiated; and the age at which cART was initiated.

3) Neuroimaging outcomes previously shown to reflect cerebral injury in our cohort: grey matter (GM) and white matter (WM) volumes; WM mean diffusivity (MD; measure of poorer WM integrity) and fractional anisotropy (FA; measure of better WM integrity) as measured with diffusion tensor imaging[8]; WM choline-to-creatine ratio (Cho:Cre; a measure of glial proliferation)[9]; and cerebral blood flow (CBF) in GM, WM, and combined subcortical regions (caudate nucleus, putamen, nucleus accumbens, thalamus).[10]

4) Cognitive outcomes previously shown to be affected in our and other pediatric cohorts: intelligence quotient (IQ), attention/working memory (AWM), processing speed (PS), and visuomotor performance (VIS).[11]

These network analyses relied on participants with complete data available on all the outcome measures chosen for the model. Hence, we could include 21 participants for the plasma-marker network analysis, and 13 participants for the CSF-marker network analysis.

Further technical details of network modeling are explained below.

Graphs: a language for networks

Networks are represented by graphs. We consider graphs $G = (V, E)$ consisting of a finite set V of vertices (or nodes) and set of edges E. The vertices of the graph correspond to a collection of random variables with a multivariate probability distribution. Edges connect pairs of vertices. We thus focus on Gaussian graphical modeling.

The support of a Gaussian precision matrix (i.e., the inverse of the covariance matrix) represents a Markov random field. This means that conditional independence between a pair of variables corresponds to zero entries in the precision matrix. Now, let $\hat{\mathbf{\Omega}}$ denote a generic estimate of the precision matrix. When the jj'th entry of the precision matrix is zero, this implies that the variables Y_j and $Y_{j'}$ are independent given the remaining variables, which implies that Y_j and $Y_{j'}$ are unconnected in the graph. Hence, model selection efforts in Gaussian graphical models focus on determining the support of the precision matrix.

To determine the support, we first need an estimate of the precision matrix. We employ a *regularized* estimate as the data can be high-dimensional in the sense of containing more variables than observations ($p > n$). Regularization will also stabilize estimates when the data are not or almost high-dimensional. The penalized Maximum Likelihood ridge estimator[3] is employed, given by:

$$\hat{\mathbf{\Omega}}^{\mathrm{I}}(\lambda) = \left\{ \left[\lambda \mathbf{I}_p + \frac{1}{4}(\mathbf{S} - \lambda \mathbf{T})^2 \right]^{1/2} + \frac{1}{2}(\mathbf{S} - \lambda \mathbf{T}) \right\}^{-1},$$

with $\mathbf{S}$ denoting the sample covariance (or correlation) matrix, where $\mathbf{T}$ denotes a symmetric positive definite target matrix, and where $\lambda \in (0, \infty)$ denotes a penalty parameter. The target matrix is taken to be the $(p \times p)$-dimensional identity matrix $\mathbf{I}_p$. The optimal penalty parameter was determined by leave-one-out cross-validation of the negative log-likelihood score. Support determination was performed based on partial-correlation thresholding (the partial correlation matrix is a scaled version of the precision matrix). The 30 strongest partial correlations were retained. The sparsified matrix then represents the network. All employed machinery is available through the package *rags2ridges* in the statistical language R.[12]

References for Supplemental Methods:

1. Goeman JJ, van de Geer SA, de Kort F, van Houwelingen HC. A global test for groups of genes: testing association with a clinical outcome. *Bioinformatics*. 2004;20(1):93-9.

2. Kendall MG, Babington Smith B. The problem of m rankings. *Ann Math Stat*. 1939;10(3):275-287.

3. Van Wieringen WN, Peeters CFW. Ridge estimation of inverse covariance matrices from high-dimensional data. *Comput Stat Data Anal*. 2016;103:284-303.

4. Blokhuis C, Kootstra NA, Caan MW, Pajkrt D. Neurodevelopmental delay in pediatric HIV/AIDS: current perspectives. *Neurobehav HIV Med*. 2016;7(1):1-13.

5. McGuire JL, Gill AJ, Douglas SD, Kolson D. Central and peripheral markers of neurodegeneration and monocyte activation in HIV-associated neurocognitive disorders. *J Neurovirol*. 2015;21(4):439-448.

6. Meucci O, Fatatis A, Simen AA, Bushell TJ, Gray PW, Miller RJ. Chemokines regulate hippocampal neuronal signaling and gp120 neurotoxicity. *Proc Natl Acad Sci U S A*. 1998;95(24):14500-14505.

7. Kamat A, Lyons JLJ, Misra V, et al. Monocyte activation markers in cerebrospinal fluid associated with impaired neurocognitive testing in advanced HIV infection. *J Acquir Immune Defic Syndr*. 2012;60(3):234-243.

8. Cohen S, Caan MWA, Mutsaerts HJ, et al. Cerebral injury in perinatally HIV-infected children compared to matched healthy controls. *Neurology*. 2016;86(1):19-27.

9. Van Dalen YW, Blokhuis C, Cohen S, et al. Neurometabolite Alterations Associated With Cognitive Performance in Perinatally HIV-Infected Children. *Medicine (Baltimore)*. 2016;95(12):e3093.

10. Blokhuis C, Mutsaerts HJMM, Cohen S, et al. Higher subcortical and white matter cerebral blood flow in perinatally HIV-infected children. *Med (United States)*. 2017;96(7):e5891.

11. Cohen S, Stege JA, Geurtsen GJ, et al. Poorer Cognitive Performance in Perinatally HIV-Infected Children Versus Healthy Socioeconomically Matched Controls. *Clin Infect Dis*. 2015;60(7):1111-1119.

12. Peeters CFW, Bilgrau AE, van Wieringen WM. rags2ridges: Ridge Estimation of Precision Matrices from High-Dimensional Data. R package, version 2.2-beta. 2017.
